# *Salmonella* Infection in Nursery Piglets and Its Role in the Spread of Salmonellosis to Further Production Periods

**DOI:** 10.3390/pathogens10020123

**Published:** 2021-01-25

**Authors:** María Bernad-Roche, Alejandro Casanova-Higes, Clara M. Marín-Alcalá, Alberto Cebollada-Solanas, Raúl C. Mainar-Jaime

**Affiliations:** 1 Departamento de Patología Animal, Facultad de Veterinaria, Instituto Agroalimentario de Aragón-IA2, Universidad de Zaragoza-CITA, 50013 Zaragoza, Spain; mbernadroche@gmail.com; 2 Unidad de Producción y Sanidad Animal, Centro de Investigación y Tecnología Agroalimentaria de Aragón, Instituto Agroalimentario de Aragón-IA2, Universidad de Zaragoza-CITA, 50059 Zaragoza, Spain; casanova.alejandro89@gmail.com (A.C.-H.); cmarin@unizar.es (C.M.M.-A.); 3 Grupo de Genética de Micobacterias, Departamento de Microbiología, Medicina Preventiva y Salud Pública, Universidad de Zaragoza, 50009 Zaragoza, Spain; alberto@unizar.es; 4 Unidad de Biocomputación, Instituto Aragonés de Ciencias de la Salud (IACS/IIS Aragón), Centro de Investigación Biomédica de Aragón (CIBA), 50009 Zaragoza, Spain

**Keywords:** nursery piglets, prevalence, *Salmonella*, swine, shedding, PFGE, zoonoses

## Abstract

Few studies have focused on assessing *Salmonella* infection in the nursery and its role in further pig production periods. Mesenteric lymph nodes, intestinal content, and meat juice from 389 6-week-old male piglets intended for human consumption from five breeding farms and 191 pooled floor fecal samples from gilt development units (GDU) from the same farms were analyzed to estimate and characterize (by pulsed-field gel electrophoresis and antimicrobial resistance analyses) *Salmonella* infection. The prevalence of infection and shedding among piglets was 36.5% and 37.3%, respectively, shedding being significantly associated with infection (Odds Ratio = 12.7; CI 7.3–22.0). *Salmonella* Rissen; *S.* 4,[5],12:i:-; and *S.* Derby were the most common serotypes. A low level of *Salmonella*-specific maternal antibodies at the beginning of the nursery period suggested it was a period of high risk of infection. Resistance to 3rd- and 4th-generation cephalosporins was detected in piglet isolates although the piglets never received antibiotics, indicating they could be vectors of antimicrobial resistance. The same *Salmonella* clones were detected in piglet and GDU isolates, suggesting that infected piglets play a significant role in the infection of gilts and consequently of finishing pigs in the case of production farms. The control of *Salmonella* infection in nursery piglets may decrease the risk of abattoir and carcass contamination.

## 1. Introduction

*Salmonella* infection is quite common in pigs in the European Union [1]. It usually courses asymptomatically, but infected pigs may shed the bacterium through their feces, making them a major risk factor for carcass contamination at slaughter [2]. Since the presence of *Salmonella* during the growing–finishing phase is directly related to carcass contamination at abattoirs, main mitigation measures have been usually directed toward this production period [3,4,5,6]. However, few studies have focused on the periods previous to this one, such as the nursery, although active *Salmonella* infections have been identified in pig nurseries [7]. 

The so-called nursery is a period that comprises the time from weaning at 3–4 weeks of age to approximately 10 weeks of age (just before entering the growing unit). This is a critical production phase in which piglets are very susceptible to a variety of enteric infections. A common consequence of weaning is the modification of the piglets´ intestinal microbiota, characterized by a significant reduction in the number of lactobacilli [8], mostly due to sudden changes in their diet, which goes from mostly liquid (sow milk) during lactation to a solid-based diet (prestarter feed) at the beginning of the nursery, and environment [9]. These changes, along with the decay of maternal antibodies [10], the only immune protection at this age, and the usual animal stress linked to the piglet’s separation from its dam and its commingling with new piglets, make weaned piglets highly prone to Gram-negative bacterial infections, such as *Escherichia coli* and *Salmonella* spp. [11].

While *E. coli* infection has been widely studied and is confirmed as a prevalent enteric pathogen at this age [12], the prevalence of *Salmonella* infection has been barely studied. However, it is accepted that weaning- or post-weaning-age pigs would be among the most clinically affected had they become infected by *Salmonella* [2]. Both are closely related bacteria that are susceptible to the same class of antibiotics, so the treatments against colibacillosis may be hindering *Salmonella* infections at this age. Indeed, until recently, the use of antimicrobials as prophylactics, mostly aminoglycosides and polymyxins (colistin), was a common practice in intensive pig husbandry systems, particularly during the nursery period [13,14]. In Spain, colistin was commonly administered, for as long as 15 days, as an in-feed antimicrobial for years, due to its high efficacy against Gram-negative bacteria [15].

In a recent study, we observed that *Salmonella* prevalence in suckling piglets from seropositive breeding farms was high, confirming that apparently healthy 4-week-old (wo) piglets may act as carriers of infection at least until weaning [16]. Considering the likely high proportion of *Salmonella*-infected piglets at weaning, it is likely that the nursery will be heavily infected by this pathogen as well. Therefore, it seems logical to think that during the past few years, *Salmonella* infection has been overlooked during the nursery period due to preventive antimicrobial treatments.

The alarming increase in antimicrobial resistance triggered the European health authorities to set up new European Union regulations on the use of colistin in veterinary medicine, and since 2015, colistin has been banned for use as a prophylactic [14]. These new regulations may favor an increasing incidence of salmonellosis in the nursery. However, to the best of the authors´ knowledge, no field studies on *Salmonella* prevalence during the nursery period have been carried out so far. Thus, in this study, we first assess the prevalence of *Salmonella* infection at the beginning of the nursery period, i.e., two weeks after weaning, in a piglet population that came from a group of *Salmonella*-seropositive breeding farms. Further, we characterize by pulsed-field gel electrophoresis (PFGE) and antimicrobial susceptibility analyses the *Salmonella* isolates obtained and compare them to those isolated from gilt development units (GDU) from the same herds. Results from this study may help to shed some light on the role that *Salmonella* infection in nursery piglets may play in subsequent production periods, such as growing/finishing, and therefore in abattoir and carcass contamination.

## 2. Results

### 2.1. Salmonella Isolation, Serotyping, and Serology in Piglets

A total of 389 weaned piglets were sampled from *Salmonella*-seropositive breeding farms (an average of 78 piglets per farm). Piglets were sampled in all seasons (35.7% in winter, 23.4% in spring, 22.4% in summer, and 18.5% in autumn). Table 1 shows the distribution of the sampling by farm and the corresponding prevalence of infection (*Salmonella*-positive in mesenteric lymph nodes) and shedding (*Salmonella*-positive in intestinal content). The prevalence of infection varied significantly between farms, ranging from 17.5% (farm E) to 59.2% (farm D), with an average of 36.5% (95% CI 31.9–41.4). The prevalence of shedding piglets also varied significantly between farms, with a similar mean value (37.3%; 95% CI 32.6–42.2).

A median of 14.4 g (95% CI 13.96–15.00) of MLN was collected. No significant difference was observed between the weight of MLN-positive and MLN-negative samples (median of 15.0 and 14.1 g, respectively; *p* = 0.31). 

All *Salmonella* isolates (145 from IC samples and 142 from MLN samples) were serotyped. The distribution of *Salmonella* serotypes by farm and type of sample is shown in Table 2. *Salmonella* Rissen was the most frequent serotype (42.8%) recovered from IC samples, followed by the monophasic variant of *S.* Typhimurium (*S.* 4,[5],12:i:-) (40.0%), and *S.* Derby (4.8%). A similar distribution of serotypes was observed in MLN-positive samples, with *S.* 4,[5],12:i:- (40.8%) and *S.* Rissen (31.0%) being the most prevalent, followed by *S.* Brandenburg (10.6%). *Salmonella* 4,[5],12:i:- was the only serotype present in all farms.

*Salmonella* was not detected in 206 (52.9%) of the sampled piglets, while positive results in both MLN and IC samples were obtained for 104 (26.7%) of them. The same serotype was detected in 86 (82.7%) of the animals with positive MLN and IC cultures (Table 2). A significant association between the isolation of *Salmonella* in MLN and IC samples was observed: an MLN-positive piglet had around 12 times higher odds of shedding *Salmonella* than an MLN-negative piglet (OR = 12.7; CI 7.3–22.0; *p* < 0.001) once the season and farm effects were accounted for (Table 3).

Regarding serological results, the median optical density percentage (OD%) value for the 389 sampled animals was 4.7 (95% CI 3.0–6.0). No significant differences were observed between MLN-negative and MLN-positive piglets (median of 4.8 and 4.7, respectively; *p* = 0.5). No significant differences were observed either in OD% values between IC-negative and IC-positive piglets (median of 5.2 and 4.0, respectively; *p* = 0.1).

### 2.2. Salmonella Isolation, Serotyping, and Serology in Gilt Development Units (GDU)

A total of 191 pooled floor fecal samples from 5 GDU were collected. *Salmonella* was isolated from 51 of them (26.7%; 95% CI 20.9–33.4), but the proportion of positive samples in the growing units varied among farms, ranging from 17.5% to 43.8% (Table 4). The most frequent serotype was *S*. Rissen (70.6%), which was present in all farms (A, B, C, D, and E), followed by *S*. Derby (17.6%; farms C and D) and *S*. 4,[5],12:i:- (5.9%; farms A, B, and E). In all the farms, except farm D, the serotypes found in the GDU were also detected in the nursery piglets (Table 2). Thus, the same serotypes, i.e., *S*. 4,[5],12:i:- and *S*. Rissen in farm A; *S*. 4,[5],12:i:-, *S*. Rissen, and *S*. Brandenburg in farm B; *S*. Rissen and *S*. Derby in farm C; and *S*. 4,[5],12:i:- and *S*. Rissen in farm E, were observed in piglets and floor samples from gilt units (Table 2). 

Four hundred and twenty-one gilt serum samples were available from four farms (A, B, C, and E; Table 4). The mean seroprevalence was 24.2% (95% CI 20.4–28.5). Among gilt units, seroprevalence varied slightly (from a minimum of 19.3% in farm E to a maximum of 31.9% in farm A).

### 2.3. Pulsed-Field Gel Electrophoresis (PFGE)

PFGE analysis was performed when the same *Salmonella* serotype was detected in piglets and floor fecal samples from gilt units from the same farm. Thus, a total of 37 *Salmonella* isolates from IC-positive piglets (15 from farm A, 9 from farms B and C, and 4 from farm E) and 17 from the GDU (5 from farm A, 2 from farm B, 6 from farm C, and 4 from farm E) were submitted for PFGE analysis. Isolates of *S*. Brandenburg from the gilt unit of farm B were not available. 

**Figure 1:**
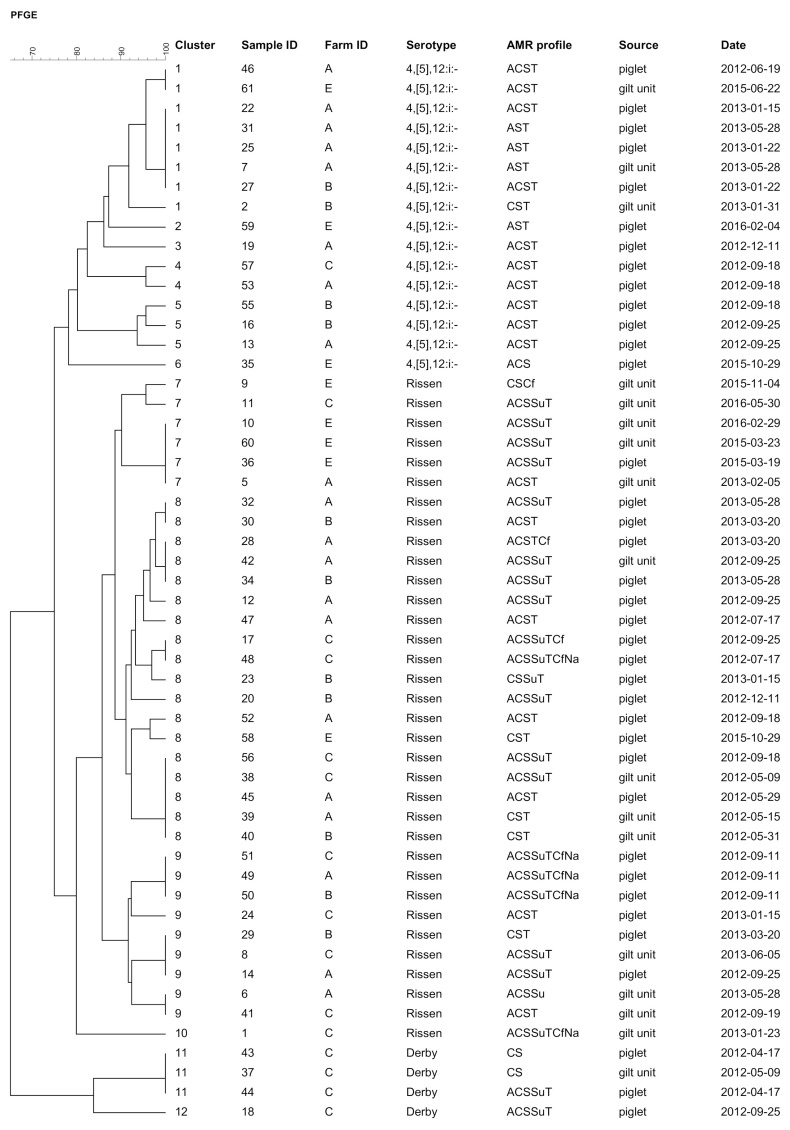
Dendrogram showing the main *XbaI* pulsed-field gel electrophoresis (PFGE) patterns (≥90% homology) for 54 *Salmonella* strains isolated from piglets´ intestinal content and GDU from 4 farms (A, B, C, and E). A, aminopenicillins; C, phenicols; S, aminoglycosides; Su, sulfonamides; T, tetracyclines; Na, quinolones; Cf, 3rd- or 4th-generation cephalosporins.

PFGE analysis of these 54 *Salmonella* isolates showed 12 different *XbaI* patterns (based on a similarity cutoff of ≥ 90%) (Figure 1). Observed PFGE clusters matched well with serotypes and antimicrobial resistance (AMR) profiles. When analyzed by serotype, six main clusters were observed for *S*. 4,[5],12:i:-, four for *S*. Rissen, and two for *S*. Derby. 

*Salmonella* isolates from the GDU were grouped into six different PFGE patterns, and within five of them isolates from piglets from the corresponding farm were included (patterns 1, 7, 8, 9, and 11; Figure 1). Overall, 75.7% of the piglet isolates analyzed were included within these five clusters. At least one genetic relationship between *Salmonella* isolates from piglet fecal samples and floor fecal samples from the corresponding GDU was detected in all four farms.

In the case of isolates from piglets, genetically similar serotypes were detected on several occasions within the same farm and sometimes more than 150 days apart (i.e., *S*. 4,[5],12:i:- and *S*. Rissen in farm A, *S*. Rissen in farm B, and *S*. Rissen and *S*. Derby in farm C). 

When looking at pairs of homologous *Salmonella* isolates coming from piglets and GDU, isolates of five of these pairs were collected within a short (<1 month) period of time, namely one *S*. Rissen pair from farm A, one *S*. 4,[5],12:i:- from farm B, one *S*. Rissen and one *S*. Derby from farm C, and one *S*. Rissen from farm E. In another five pairs, the piglet isolates were collected far before (>150 days before) the gilt samples (one *S*. Rissen from farm A, one *S*. 4,[5],12:i:- from farm B, one *S*. Rissen and one *S*. Derby from farm C, and one *S*. Rissen from farm E). In two more (one *S*. Rissen from farm B and one from farm C), the piglet isolates were collected far after gilt samples had been obtained (Figure 1).

### 2.4. Antimicrobial Resistance

All 54 isolates submitted to PFGE were also tested for AMR against 17 antimicrobial agents, as described below. All but two *S*. Derby displayed multidrug resistance (MDR), both isolated from farm C (Table 5). A total of 11 multi-AMR profiles were detected, the most common being ACST (*n* = 17 31.5%) and ACSSuT (*n* = 15, 27.8%), both of them mostly associated with *S*. Rissen and detected in all the farms (Figure 1). The most common phenotypic resistance was against florfenicol (92.6%), tetracycline (90.7%), ampicillin (83.3%), and trimethoprim-sulfamethoxazole (42.6%). 

Regarding resistance to antimicrobials of critical importance for humans, no AMR against carbapenems (imipenem) or polymyxins (colistin) was detected in any of the isolates analyzed. However, AMR against cephalosporins of 3rd (ceftiofur) and 4th (cefquinome) generations was detected in 13.0% (7/54) and 5.6% (3/54) of the isolates, respectively. All of them were *S*. Rissen.

## 3. Discussion

To properly assess the true prevalence of *Salmonella* infection in nursery units at a pig farm is quite challenging and expensive, as it requires the killing of a large number of young animals (between 4 and 10 weeks of age) that are usually intended for other purposes, i.e., either for slaughter at market age (5–6 months old) or as replacement animals. For this study, piglets came from five breeding farms where female weaned piglets were raised as re-stocking gilts for other pig production farms, while male piglets were fattened up to 7–9 kg live weight and then slaughtered for human consumption. These male piglets were weaned when they were 4 wo and fed for two more weeks, until slaughtering. This management allowed us to analyze MLN, IC, and meat juice samples in order to obtain a good assessment of the true *Salmonella* incidence status of these piglets. In addition, these piglets came from *Salmonella*-seropositive breeding herds [16] and had not been treated with antibiotics, as they were to be slaughtered. Thus, this approach could somewhat reflect what may be happening in nursery piglets from *Salmonella*-seropositive production herds.

The overall percentage of *Salmonella*-infected (MLN-positive) piglets was 36.5% (95% CI 31.9–41.4) but was variable across pig farms (from 17.5% to 59.2%). A similar proportion of piglets (37.3%; 95% CI 32.6–42.2) could be considered *Salmonella* shedders as the bacterium was found in their IC (Table 1). Few field studies have been carried out on *Salmonella* prevalence at this stage, and when performed through the sampling of live piglets, results suggested active infection but the recovery levels of fecal *Salmonella* were usually much lower [7,17]. Two major issues would help to understand the differences between this and previous studies. First, we analyzed 25 g of fecal content, the amount required according to the ISO 6579:2002/A1:2007 standard and significantly larger than that collected through swabbing. Second, this study only included piglets from breeding farms with high levels of *Salmonella* seropositivity. A good match was also found between animal infection and shedding at this age, with the odds of shedding *Salmonella* being more than 12 times higher for an MLN-positive piglet compared to an MLN-negative one (Table 3). This relationship was further supported by the detection of the same *Salmonella* serotype on MLN and IC in most (82.7%) of the piglets that had been positive in both types of samples. Shedding the bacterium through feces is the main mechanism of transmission of *Salmonella* and is common when animals are infected for the first time or when infected animals suffer episodes of stress, such as commingling or transport [11]. Given the age of these piglets, they had probably been infected very recently, besides suffering such stress factors as they had been sent to slaughter. In addition, an unknown number of new infections may have also occurred during transport and/or lairage [18,19]. Altogether, this would help to explain the high level of shedding among these piglets. From these results, it can be derived that most of the post-weaning pigs that become infected with *Salmonella* are also asymptomatic, and in contrast to what was expected [2], it appears that when compared to older piglets, they would not be more clinically affected. Thus, salmonellosis may be easily overlooked during the nursery period as well, regardless of the use of antimicrobials. In any case, this high prevalence of infection and shedding was not a surprising result, since similar findings were observed in a previous study on weaned (4-wo) pigs from the same farms [16]. 

No differences were found regarding serological OD% values between infected and non-infected piglets (median OD% of 4.7 and 4.8, respectively), these values being similar to those found in piglets of the same age in other studies [20]. However, on average, OD% values were much lower than those found in the previous study on 4-wo piglets from the same farms (15.9%; [16]), evidencing the likely significant decay of maternally derived IgGs against *Salmonella* within the two weeks after weaning [7]. It seems that the beginning of the nursery may be a period of high susceptibility to the infection and that measures to prevent exposure to *Salmonella* should be encouraged at this time. 

One of the aims of this study was to determine whether *Salmonella* infection from nursery units will be able to reach further production phases, that is, the growing units. For that purpose, floor fecal samples from the GDU, from each of corresponding breeding farms under study, were collected in order to detect *Salmonella*. All the gilt units were *Salmonella* positive, with an overall proportion of positive samples of 26.7% (Table 4). This figure would be within the expected range of *Salmonella*-positive fecal samples for growing/finishing pig units in Spain [1,21] but probably underestimates the true proportion of positive samples, given the relative low sensitivity of the bacteriological culture when performed on fecal samples from asymptomatic animals [5].

The main serotypes detected in these gilt units were *S.* Rissen, followed by *S*. Derby and *S*. 4,[5],12:i:-. The three of them were also the most common serotypes detected in the piglets, suggesting that these serotypes were circulating between both production periods even when the GDU were located kilometers away from their corresponding nurseries (i.e., farm E). To further confirm this hypothesis, PFGE analysis was performed on *Salmonella* isolates when the same serotype was found in piglets and gilt units from a given farm. PFGE analysis showed that most of the *Salmonella* isolates from piglets were grouped with gilt isolates, indicating a high level of genetic similarity (≥90%) between them. Despite the small number of isolates analyzed, this match was found at least once in all four farms where these isolates were recovered and it was detected for the three serotypes, supporting the maintenance of *Salmonella* infection between the nursery and the growing unit and the role that piglets may play in it. 

Within a given nursery unit, the same clone of *Salmonella* could be detected for a long period of time (>150 days apart) involving different batches of piglets. Besides, no particular temporal pattern of infection between piglets and gilt units was detected either (a given *Salmonella* isolate could be found at the same time or first in nursery piglets and later in the gilt units, or vice versa). These results suggested that cross-contamination between units within farms and/or a lack of proper pen hygiene are major issues in these farms, highlighting the difficulties of eliminating *Salmonella* from the farm environment despite high internal hygiene and biosecurity standards. These results would also help to explain, in part, the origin of the *Salmonella* infection in the GDU and the further *Salmonella* infection in sow herds [22].

It is worth noting that *S.* Derby appears as one of the most frequent serotypes found in breeding farms (~30%) [23] and is commonly found throughout the pig production pyramid, which would support the hypothesis that this serotype is easily transmitted by the transfer of animals between units or herds [24]. With regard to *S.* Rissen and *S*. 4,[5],12:i:-, both were also reported in the European survey mentioned above and both have experienced a worldwide expansion in the past few decades [25,26]. In a recent report from the Spanish Ministry of Agriculture, Fish and Food, the monophasic variants of *S*. Typhimurium and *S*. Rissen were the most prevalent serotypes found in a national survey carried out on slaughtered pigs in 2019 (35.4% and 23.8%, respectively), followed by *S*. Derby (13.1%) [27]. Both *S.* Derby and *S*. 4,[5],12:i:- are among the main pig-related serotypes associated with human salmonellosis in the EU in the past few years [28,29,30,31,32], while *S.* Rissen is considered a significant cause of foodborne salmonellosis in Asia and southern European countries [26,33]. To the best of the authors’ knowledge, there are no reports so far on the main *Salmonella* serotypes associated with contamination of piglet meat or showing possible links between consumption of piglet meat and human salmonellosis. However, considering these results, health authorities should be aware of the potential risk of contamination of piglet carcasses due to the arrival of infected piglets at the abattoir.

Ninety-eight percent of the *Salmonella* isolates displayed multidrug-resistant phenotypes, with most of them showing resistance profiles commonly reported in *Salmonella* (ACSSu, ACSSuT, ACST, etc.). No AMR was found against antibiotic classes considered of critical importance for humans, such as carbapenems and polymyxins [34], but a significant proportion of isolates (12.9%) presented resistance to 3rd-generation cephalosporins and even to cefquinome (5.5%), a 4th-generation cephalosporin. This type of resistance, detected only in *S.* Rissen isolates, was much more prevalent than that reported in pigs for the EU (1.1%) [35]. The worldwide emergence of resistance to extended-spectrum cephalosporins in non-typhoidal *Salmonella* is already a matter of concern [36], particularly when harbored by *Salmonella* serotypes of potential zoonotic character [37]. In this case, most of the resistant isolates (75%) were found in piglets intended for human consumption. Thus, they could be considered potential vectors for the transmission of this resistance to humans, although they never received antibiotics.

In summary, nursery pigs can become subclinically infected and act as active carriers of *Salmonella* in a farm, as the same *Salmonella* clones were observed in piglets and in the GDU. This finding suggests that piglets play a significant role in the infection of finishing pigs in production farms as well. Therefore, *Salmonella* infection in nursery piglets should be considered a potential risk factor for abattoir contamination, and the control of *Salmonella* infection at this stage may help to decrease the risk of carcass contamination. Considering that a significant source of *Salmonella* for nursery pens would be the sows [16], sows would then be a significant source of *Salmonella* infection for gilts and finishing pigs [38,39]. Indeed, a recent risk assessment model adapted specifically for Spain showed that sow prevalence is a strong indicator of slaughter pig prevalence [40]. Our results support theirs. Defining proper strategies that prevent *Salmonella* shedding from sows should be a major goal in any production/breeding pig farm. Factors that could be related to *Salmonella* infection/shedding in sows (i.e., dry/pellet feed, inefficient disinfection protocols, type of floor, etc.) [39,41,42] should then be avoided. The implementation of feed strategies (organic acids, essential oils, prebiotics, etc.) to reduce somehow the level of infection and shedding in the farms [43,44], or even the vaccination of sows [45,46], may also be advisable. These strategies could be implemented directly in nursery piglets as well [47,48]. In addition, minimizing the use of antibiotics in production/breeding farms would appear as another goal to prevent further spread of AMR to fattening pigs and gilts [26] and may reduce *Salmonella* shedding as well [49].

## 4. Materials and Methods

### 4.1. Farm Selection

The study was carried out on five multiplier/supplier swine breeding farms (herd size between 700 and 940 sows) from the northeast part of Spain (A, B, C, D, and E), where half of the Spanish pig census is concentrated. Farms A, B, and C belonged to one company and D and E to a different one. All these farms had shown *Salmonella* seroprevalence ≥50%, and *Salmonella* was detected, on average, in 22% of the sow fecal samples analyzed from them [16]. The farms were chosen due to the farmers´ and corresponding veterinarians´ willingness to participate in the study. The sampling was carried out in two periods, between January 2012 and May 2013 (farms A, B, and C) and between February 2015 and February 2016 (farms D and E). 

These breeding farms keep female weaned piglets as re-stocking gilts for other pig production farms. However, male weaned piglets are usually sent to slaughter for meat, either at weaning age (4 wo) or after a period of two weeks in the nursery (6 wo). The samples analyzed in this study belonged to 6-wo male piglets.

### 4.2. Collection of Samples

Piglet samplings were obtained at the abattoir. On a given day, whole intestinal packages from a certain number of animals from a given batch from a farm were collected directly at the slaughter line. There was no routine sampling schedule as the number of available piglets and sampling times were dependent upon both piglet availability from any of the five selected farms and abattoir readiness for collaboration that day. The intestinal packages were then submitted to the laboratory for immediate processing. The maximum-possible amount of mesenteric lymph nodes (MLN) and a minimum of 25 g of intestinal content (IC) were collected for bacteriological analysis. A piece of the diaphragm muscle was also collected for serological analysis.

During the period of piglet sampling, and every 3–4 months, floor fecal samples were also collected by farm veterinarians from GDU from the corresponding farms. In addition, serum samples from a representative number of these gilts were available for that period from the official eradication campaign against Aujeszky´s disease. Fecal and serum samples were useful in identifying circulating *Salmonella* serotypes during the growing period and assessing the serological status of the growing gilts, respectively. 

### 4.3. Bacteriology

Detection of *Salmonella* isolates in both IC and MLN samples was performed according to the ISO 6579:2002/A1:2007 standard. Briefly, fresh MLN samples were first defatted, weighed, and externally decontaminated by dipping them into absolute alcohol and further flaming them. Afterward, the samples were homogenized in buffered peptone water (BPW; Panreac Quıímica SAU, Castellar del Vallés, Spain) in 1:10 dilution and incubated for 18 ± 2 h at 37 ± 1 °C. Thereafter, three drops (33 µL each) of incubated BPW were inoculated into a modified semisolid Rappaport Vassiliadis medium (MSRV; Oxoid Ltd., Hants, UK), and plates were incubated for 24 ± 3 h at 41.5 ± 1 °C (negative samples were re-incubated for an additional 24 h). Ten microliters of the presumptive *Salmonella* growth (detected by the halo generated in MSRV after 24 or 48 h) was transferred to two selective media (xylosine lysine deoxycholate (XLD) and brilliant green (BG) agars) (Panreac Quıímica SAU, Castellar del Vallés, Spain). One suspected colony per plate from each *Salmonella*-positive MLN and IC sample was confirmed biochemically (triple sugar iron (TSI) agar, urea agar, L-lysine decarboxylation medium, and indole reaction) (Panreac Quıímica SAU, Castellar del Vallés, Spain) and further serotyped (sera obtained from the Statens Serum Institut, Copenhagen, Denmark) at the National Reference Laboratory for Animal Salmonellosis in Madrid, Spain, following the White–Kauffmann–Le Minor scheme [50].

### 4.4. Pulsed-Field Gel Electrophoresis Analysis

To assess the genetic relationship between the *Salmonella* strains shed by piglets and *Salmonella* contamination in the GDU, PFGE analysis was performed (CHEF-DR^®^ III System, BIO-RAD, Madrid, Spain) on *Salmonella* isolates according to the pulse net protocol [51]. This technique is broadly recognized as a sensitive method for the molecular fingerprinting of *Salmonella* serotypes and is very useful for tracing the spread of this bacterium through the different pig production phases [24].

Only isolates from piglets’ fecal samples and floor fecal samples from the gilt unit from the same farm that showed the same serotype were analyzed. If several isolates met this criterion, then a maximum of two piglet isolates and two gilt isolates per batch were analyzed.

PFGE pattern analysis was performed with the BIONUMERICS software (version 6; Applied Maths, Sint-Martens-Latem, Belgium) using Dice’s coefficient and the unweighted pair group method with arithmetic averages (UPGMA dendrogram type) with a position tolerance of 1.5% and optimization of 2.0%.

### 4.5. Antimicrobial Agent Susceptibility

AMR tests were also performed on isolates submitted for PFGE analysis in order to further characterize them. Susceptibility to aminopenicillins (A; ampicillin, amoxicillin-clavulanic acid), phenicols (C; florfenicol), aminoglycosides (S; gentamicin, neomycin), sulphonamides and dihydrofolate reductase inhibitors (Su; trimethoprim-sulfamethoxazole), tetracyclins (T; tetracycline), cephalosporins (Cf; cephalexin, cefalotin, cefoperazone, ceftiofur, cefquinome), polymyxins (Po; colistin), carbapenems (Cm; imipenem), and quinolones (Na; flumequine, enrofloxacin, marbofloxacin) was assessed by the VITEK-2 automated system with VITEK GN96 cards (BioMérieux, Marcy-l’Étoile, France). The minimum inhibitory concentration (MIC) was obtained for each *Salmonella* strain and further classified as resistant, intermediate, or susceptible according to the Clinical and Laboratory Standard Institute recommendations [52]. An isolate displaying phenotypic resistance to at least three antimicrobial classes was considered multidrug resistant [53].

For assessing colistin resistance, MICs were determined by the broth microdilution method according to the ISO 20776-1:2006 standard. An epidemiological cutoff (ECOFF) value of > 2 mg/L was used for considering microbiological resistance according to the recommendations of the European Committee on Antimicrobial Susceptibility Testing [54]. *Escherichia coli* ATCC 25922 and *E. coli* B13129OT (kindly provided by Dr. González-Zorn´s lab) were used as quality negative and positive control strains.

### 4.6. Serology

For the detection of specific antibodies (IgG) against *Salmonella* spp., meat juice (MJ) from piglets’ diaphragm muscle was used. MJ samples were obtained after freezing and further thawing a portion of the muscle. For gilts, blood serum samples were collected. Both piglet MJ and gilt serum samples were kept at –20 °C until their use. The Herdcheck Swine *Salmonella* ELISA test (IDEXX Laboratories, Westbrook, ME, USA) was used following the manufacturer´s instructions. This test targets the main swine *Salmonella* serogroups (B, C1, and D). For piglets, results were presented as OD% values. In the case of gilts, and given the expected low specificity of the ELISA test used [55,56], a cutoff value of OD% ≥ 40 was deemed to classify a gilt as seropositive.

### 4.7. Statistical Analysis

Prevalence of *Salmonella* infection and shedding among piglets and their corresponding 95% confidence intervals (95% CI) were estimated. Since the weight of MLN samples may differ among piglets and therefore may influence bacteriological results, the median MLN sample weight was compared between *Salmonella*-positive and *Salmonella*-negative piglets by means of the Mann–Whitney test for independent samples to detect potential bias. The relationship between piglet shedding and infection was assessed by mixed logistic regression after adjusting by season and considering farm as a grouping factor (gllamm module in STATA software). ELISA OD% values between *Salmonella*-infected and non-infected piglets were compared using the Kruskal–Wallis test. The software STATA (STATA/IC 12.1. Stata-Corp. LP, College Station, TX, USA) was used for statistical analyses.

## Figures and Tables

**Table 1:** Results for *Salmonella* isolation * from intestinal content (IC) and mesenteric lymph nodes (MLN) in 6-week-old nursery piglets.

Farm	No. of Piglets	No. of MLN-Positive (%)	No. of IC-Positive (%)	No. of IC- and MLN-Positive (%) **
A	96	39 (40.6)	47 (48.9)	31 (56.4)
B	75	34 (45.3)	26 (34.7)	20 (50.0)
C	89	26 (29.2)	34 (38.2)	22 (57.9)
D	49	29 (59.2)	22 (44.9)	20 (64.5)
E	80	14 (17.5)	16 (20.0)	11 (57.9)
Total	389	142 (36.5)	145 (37.3)	104 (56.8)

* International Organization for Standardization (ISO) 6579:2002/A1:2007. ** Percentage estimated from positive (either IC or MLN) piglets.

**Table 2:** Distribution of *Salmonella* serotypes in 6-week-old nursery piglets and in gilt development units (GDU) among the 5 farms.

Farm	Piglet Isolates				No. of Piglets with the Same Serotype in MLN–IC	GDU Isolates	
	IC		MLN				
	Serotype	No. (%)	Serotype	No. (%)		Serotype	No. (%)
A	Rissen	25 (53.2)	Rissen	19 (48.7)	17	Rissen	11 (78.6)
	4,[5],12:i:-	15 (31.9)	4,[5],12:i:-	17 (43.6)	9	Anatum	2 (14.3)
	Derby	4 (8.5)	Brandenburg	1 (2.6)		4,[5],12:i:-	1 (7.1)
	Kapemba	2 (4.3)	Goldcoast	1 (2.6)			
	Typhimurium	1 (2.1)	London	1 (2.6)			
B	Rissen	12 (46.2)	Rissen	8 (23.5)	5	Rissen	4 (66.6)
	4,[5],12:i:-	5 (19.2)	4,[5],12:i:-	8 (23.5)	3	Brandenburg	1 (16.7)
	Goldcoast	5 (19.2)	Goldcoast	3 (8.8)	3	4,[5],12:i:-	1 (16.7)
	Brandenburg	3 (11.5)	Brandenburg	13 (38.2)	3		
	Derby	1 (3.8)	Derby	2 (5.9)	1		
C	Rissen	23 (67.6)	Rissen	13 (50)	12	Rissen	10 (83.3)
	4,[5],12:i:-	7 (20.6)	4,[5],12:i:-	6 (23.1)	2	Derby	2 (16.7)
	Derby	2 (5.9)	Derby	5 (19.2)	1		
	Anatum	1 (2.9)	Anatum	1 (3.8)	1		
	Kedougou	1 (2.9)	Typhimurium	1 (3.8)			
D	4,[5],12:i:-	19 (86.3)	4,[5],12:i:-	18 (62.1)	15	Derby	7 (70.0)
	Goldcoast	1 (4.5)	Goldcoast	9 (31.0)	1	Rissen	3 (30.0)
	Brandenburg	1 (4.5)	Brandenburg	1 (3.4)	1		
	Ohio	1 (4.5)	Ohio	1 (3.4)	1		
E	4,[5],12:i:-	12 (75.0)	4,[5],12:i:-	9 (64.3)	8	Rissen	8 (88.9)
	Rissen	2 (12.5)	Rissen	4 (28.6)	2	4,[5],12:i:-	1 (11.1)
	Anatum	1 (6.3)	Anatum	1 (7.1)	1		
	Agona	1 (6.3)					
Total		145		142	86		51

**Table 3:** Association between *Salmonella* shedding and *Salmonella* infection in 6-week-old nursery piglets by mixed logistic regression analysis *.

	No. of Piglets	No. (%) of IC-Positive Piglets	Logistic Regression Parameters		
			OR	95% CI (OR)	*p*
MLN					
Negative ^1^	247	41 (16.6)	1	-	-
Positive	142	104 (73.2)	12.71	7.33–22.05	<0.001
Season					
Winter ^1^	139	33 (23.7)	1	-	-
Spring	91	26 (28.6)	1.17	0.55–2.51	0.672
Summer	87	56 (64.4)	2.69	1.31–5.55	0.007
Autumn	72	30 (41.7)	2.53	1.22–5.26	0.013

* Farm used as grouping factor. ^1^ Reference category. IC: intestinal content; MLN: mesenteric lymph nodes; OR: odds ratio.

**Table 4:** Results for *Salmonella* isolation * in floor fecal samples from gilt development units and *Salmonella* seroprevalence ** in gilts from the 5 farms.

Farm	No. of Floor Fecal Samples	No. of Positive Floor Fecal Samples (%) *	No. of Serum Samples	No. of Seropositive Samples (%) **
A	32	14 (43.8)	91	29 (31.9)
B	24	6 (25.0)	90	25 (27.8)
C	32	12 (37.5)	90	19 (21.1)
D	57	10 (17.5)	NA	-
E	46	9 (19.6)	150	29 (19.3)
Total	191	51 (26.7)	421	102 (24.2)

* ISO 6579:2002/A1:2007. ** Considering a cutoff value of OD% ≥ 40% (Herdcheck Swine *Salmonella* ELISA test, IDEXX Laboratories, USA); NA: not available.

**Table 5:** Antimicrobial resistance (AMR) patterns found in the 54 *Salmonella* isolates from 6-week-old nursery piglets and gilt development units.

AMR Family Pattern *	No. of Strains	Serotypes Involved (No. of Strains)	Farm
ACS	1	4,[5],12:i:-	E
ACSSu	1	Rissen	A
ACSSuT	15	Rissen (13), Derby (2)	A, B, C, E
ACSSuTCf	1	Rissen	C
ACSSuTCfNa	5	Rissen	A, B, C
ACST	17	4,[5],12:i:- (10), Rissen (7)	A, B, C, E
ACSTCf	1	Rissen	A
AST	4	4,[5],12:i:-	A, E
CS	2	Derby	C
CSCf	1	Rissen	E
CSSuT	1	Rissen	B
CST	5	Rissen (4), 4,[5],12:i:- (1)	A, B, E

* A, aminopenicillins; C, phenicols; S, aminoglycosides; Su, sulfonamides; T, tetracyclines; Na, quinolones; Cf, 3rd- or 4th-generation cephalosporins.

## Data Availability

Data available upon request.
